# Temporal Trends and Regional Disparities in Knee Osteoarthritis Across the United Kingdom: Insights From the Global Burden of Disease Study 1990–2021

**DOI:** 10.7759/cureus.92499

**Published:** 2025-09-16

**Authors:** Muhammad Tayyab, Zawar Ahmad, Muhammad Tanveer, Mahmood Ahmad, Suleman Shah, Rahman Syed, Ameer Afzal Khan, Muhammad Shabir, Mohsin Ali, Mohammad Idrees

**Affiliations:** 1 Department of Trauma and Orthopaedics, Bradford Teaching Hospitals, Bradford, GBR; 2 Department of Trauma and Orthopaedics, Kettering General Hospital, Kettering, GBR; 3 Department of Orthopedics, Royal Stoke Hospital, North Midlands, GBR; 4 Department of Truama and Orthopedics, Milton Keynes University Hospital, Milton Keynes, GBR; 5 Department of Nursing, Fatima College of Health Sciences, Al Ain, ARE; 6 Department of Internal Medicine, Swat Medical College, Swat, PAK; 7 Department of Internal Medicine, Saidu Medical College, Swat, PAK; 8 Department of Orthopaedics, Saidu Group of Teaching Hospital, Swat, PAK

**Keywords:** disability-adjusted life years (dalys), global burden of disease 2021, knee osteoarthritis (koa), prevalence, united kingdom (uk), ylds

## Abstract

Background and aim

Knee osteoarthritis (OA) is a major cause of pain, disability, and reduced quality of life, with its burden rising due to ageing, obesity, and lifestyle changes. This study analysed the trends between 1990 and 2021 in the prevalence, incidence, years lived with disability (YLDs), and disability-adjusted life years (DALYs) for knee OA across the four UK nations (England, Northern Ireland, Scotland, and Wales) using Global Burden of Disease data.

Methods

We extracted age-standardised and absolute estimates of prevalence, incidence, YLDs, and DALYs for knee OA in the UK and its four nations from the GBD 2021 database. Joinpoint regression analysis was applied to quantify annual percent change (APC) and average annual percent change (AAPC) in disease burden over time. Temporal patterns and inter-regional differences were assessed.

Results

From 1990 to 2021, the knee OA burden increased across all UK nations. England had the highest absolute prevalence, with knee OA rising from 3.02 million to 4.51 million cases (+49.2%), while Northern Ireland showed the largest relative increase (+76.4%). Women consistently had 30%-40% higher rates than men, with 2021 prevalence reaching 5,226.8 per 100,000 in England versus 3,753.2 in men. YLD rates rose by 7.16% in England and 7.94% in Scotland. DALYs increased most in Scottish men (+11.0%), and Northern Ireland recorded the fastest prevalence growth. England’s burden peaked around 2015 before stabilising.

Conclusion

The burden of knee OA in the UK has risen markedly over the past three decades, with persistent regional disparities. These findings highlight the need for targeted prevention strategies, early diagnosis, and equitable access to joint-preserving interventions. Public health policies addressing obesity, promoting physical activity, and improving OA management could help mitigate future disease burden.

## Introduction

Osteoarthritis (OA) is the most common form of arthritis and a leading cause of disability worldwide, characterised by progressive degeneration of articular cartilage, subchondral bone remodelling, and synovial inflammation [1]. Knee OA is the most common subtype of OA-related impairment due to the joint's crucial involvement in movement and weight-bearing [2]. The Global Burden of Disease (GBD) 2021 study indicates that approximately 600 million people worldwide are affected by OA, with knee involvement accounting for more than 80% of the total disease burden [3].

Knee OA is a significant public health issue caused by a number of interconnected variables, including an ageing population, increased incidence of obesity, sedentary lifestyles, and occupational exposures [4,5]. The condition is substantially age-related, with frequency rising sharply after middle age, notably among people over 50 [6]. Excess body weight not only increases the mechanical load on the knee joint, but it also stimulates inflammatory processes that speed up disease progression [7]. Furthermore, lifestyle changes in recent decades, such as less physical activity and more sedentary behaviour, have exacerbated these risks [8].

In the United Kingdom, OA is a major source of healthcare utilisation, job absenteeism, and loss of productivity, leading to significant economic consequences [9]. It is also the principal indication for knee replacement surgery, the most common elective orthopaedic treatment in the UK [10]. Despite its growing importance, there has been little comprehensive long-term analysis of the knee OA burden in the four UK nations - England, Scotland, Wales, and Northern Ireland - particularly in terms of prevalence, incidence, years lived with disability (YLDs), and disability-adjusted life years (DALYs).

The GBD framework provides a unique opportunity to investigate long-term epidemiological trends in knee OA by combining data from diverse sources and generating standardised estimates throughout time and regions. Understanding these trends is critical for developing focused prevention programmes, allocating resources, and determining healthcare policy. This study aimed to assess temporal trends in the prevalence, incidence, YLDs, and DALYs of knee OA in the United Kingdom between 1990 and 2021, with particular attention to regional variations and implications for public health planning.

## Materials and methods

Overview

The Institute for Health Metrics and Evaluation (IHME) carried out the Global Burden of Disease (GBD) 2021 study, which offers thorough and comparable estimates of the burden of illnesses, injuries, and risk factors in 204 nations and territories between 1990 and 2021 [11]. In order to generate internally consistent estimates across time and between regions, the GBD framework incorporates information from epidemiological studies, hospital records, outpatient registries, and national surveys into a standardised modelling system [12]. In this study, we evaluated the regional differences and temporal changes in the burden of knee OA in the United Kingdom and its four member nations (Northern Ireland, Wales, Scotland, and England) between 1990 and 2021. The publicly accessible GBD Results Tool was used to estimate incidence, prevalence, and DALYs [13].

Case definition and data sources

The GBD 2021 study classified knee OA as radiographically confirmed symptomatic Kellgren-Lawrence (KL) grade 2-4 disease [14]. Those with grades 3-4 have osteophytes, joint space narrowing, deformity, and persistent discomfort, whereas those with grade 2 have osteophytes and pain for at least one month in the previous year [15]. The burden of knee osteoarthritis was calculated using data from published epidemiological studies that met the GBD inclusion criteria, hospital discharge records, outpatient visit data, and population-based surveys [12].

Estimation of prevalence, incidence, and DALYs

The GBD estimation procedure generates age-, sex-, and location-specific estimates of incidence, prevalence, and YLDs using the Bayesian meta-regression tool DisMod-MR 2.1 (Institute for Health Metrics and Evaluation (IHME), University of Washington, Seattle, WA, USA) [12,16]. DALYs are equal to years of life lost (YLLs), and as knee OA is not a deadly condition, YLLs are set to zero. The direct technique and the World Health Organization's standard population were used to determine age-standardised rates (ASRs) per 100,000 people [12].

Statistical analysis

Joinpoint regression analysis (Joinpoint Regression Program, Version 5.4.0, National Cancer Institute, Bethesda, MD, USA) was used to evaluate the temporal trends in age-standardised incidence, prevalence, and DALY rates from 1990 to 2021 [17]. This method computes the annual percentage change (APC) and average annual percentage change (AAPC) with 95% confidence intervals (CI), identifying when statistically significant shifts in trends occur. A positive APC or AAPC with a lower CI bound above zero indicates an increasing trend, while a negative APC or AAPC with an upper CI bound below zero shows a decreasing trend. The ASRs of England, Scotland, Wales, and Northern Ireland were compared to assess the regional differences. Statistical significance was determined at p<0.05.

Inclusion and exclusion criteria

In this study, we included data on OA as defined by the GBD 2021 methodology, which incorporates symptomatic disease confirmed by clinical or radiographic evidence of joint degeneration [14]. The analysis was restricted to the four constituent countries of the United Kingdom (England, Scotland, Wales, and Northern Ireland) and covered the period from 1990 to 2021. Both sexes and all age groups were considered, and outcomes included age-standardised incidence, prevalence, years lived with disability (YLDs), and DALYs. We excluded data pertaining to other forms of OA, such as hip, hand, or generalised OA, to maintain disease specificity. Additionally, estimates not derived from the standardised GBD 2021 framework or those lacking age-standardised rates or country-level stratification were excluded. Data from outside the United Kingdom were also not considered.

Ethical considerations

This study used publicly available, de-identified secondary data from the GBD database and did not require institutional ethical approval.

## Results

Prevalence

**Figure 1:**
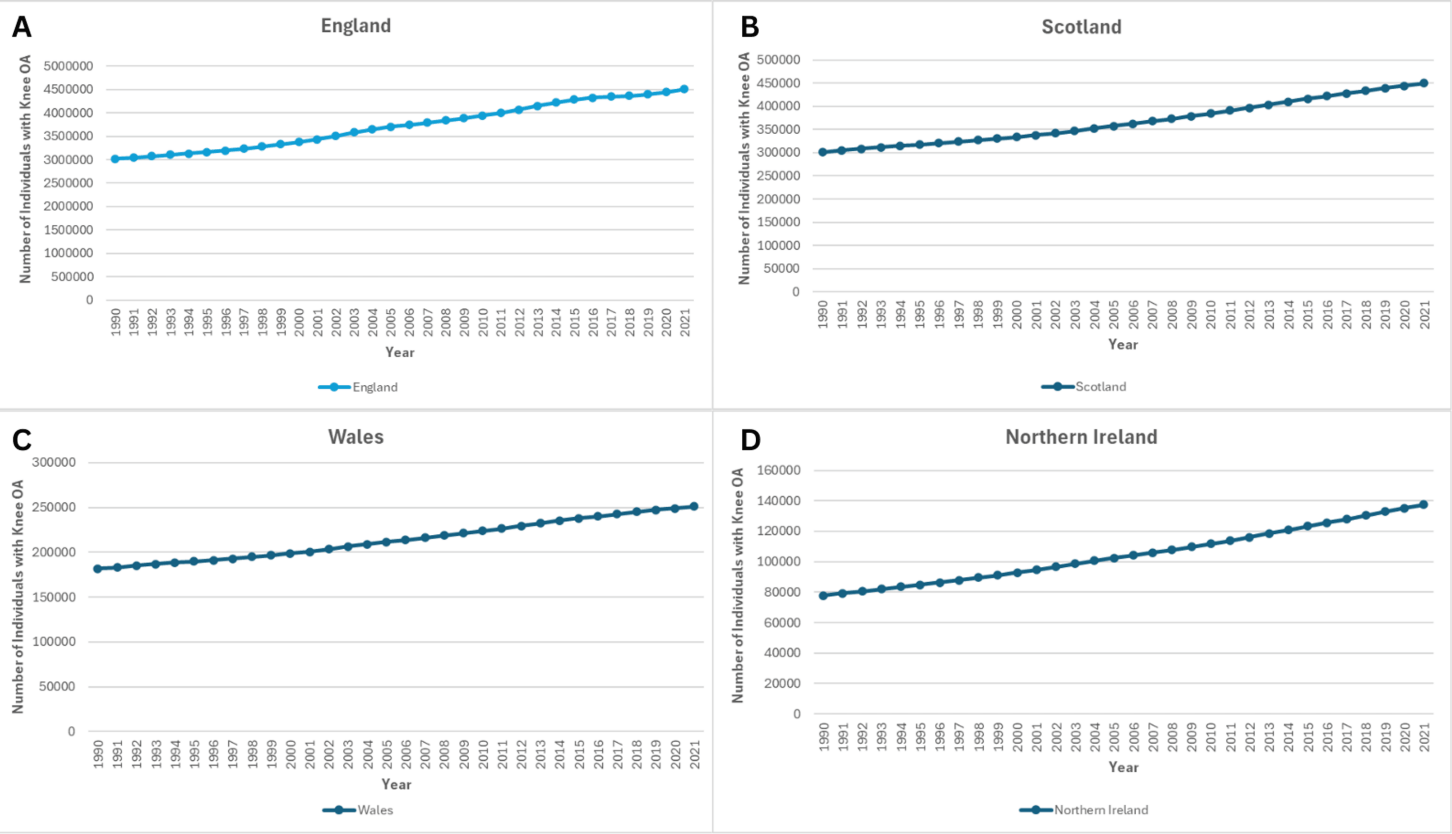
Trends in the Prevalence of Knee Osteoarthritis in the United Kingdom, from 1990 to 2021 Panel A: Prevalence in England. Panel B: Prevalence in Scotland. Panel C: Prevalence in Wales. Panel D: Prevalence in Northern Ireland. Prevalence of knee osteoarthritis in the United Kingdom from 1990 to 2021, stratified by country. The figure demonstrates temporal trends highlighting regional variations.

Between 1990 and 2021, the prevalence of knee OA increased across all four UK nations. In England, cases rose from 3,021,951 (95% UI: 2,626,557-3,470,953) in 1990 to 4,508,877 (95% UI: 3,914,575-5,145,747) in 2021, a 49.2% increase. Scotland experienced a similar rise, from 301,190 (95% UI: 259,406-346,829) to 449,459 cases (95% UI: 389,775-514,030) (+49.2%). Wales recorded an increase from 181,604 (95% UI: 155,679-209,039) to 251,143 cases (95% UI: 218,801-288,612) (+38.3%), while Northern Ireland showed the largest proportional growth, from 77,855 (95% UI: 67,388-89,680) to 137,364 cases (95% UI: 119,762-157,484) (+76.4%), as shown in Figure 1.

**Figure 2:**
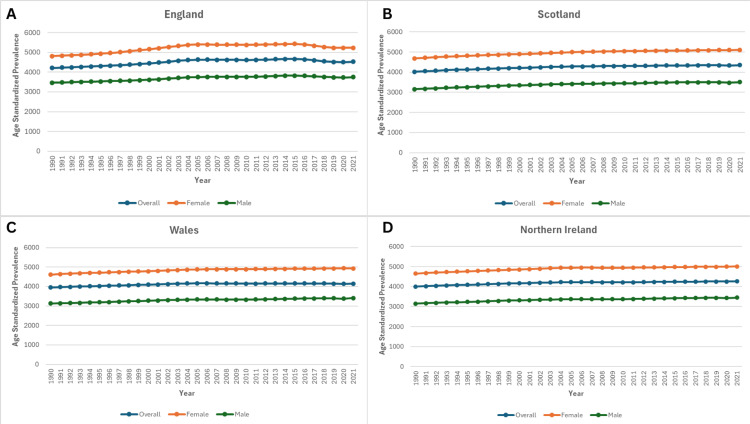
Trends in Age-Standardised Prevalence of Knee Osteoarthritis in the United Kingdom, from 1990 to 2021. Panel A: Age-standardised prevalence in England. Panel B: Age-standardised prevalence in Scotland. Panel C: Age-standardised prevalence in Wales. Panel D: Age-standardised prevalence in Northern Ireland. Age-standardised prevalence rates of knee OA in the United Kingdom from 1990 to 2021, stratified by country. The figure demonstrates temporal trends highlighting regional variations.

Age-standardised prevalence rates also rose over the study period, although the magnitude varied by region. In England, the rate increased from 4,209.8 per 100,000 (95% UI: 3,661.2-4,808.6) in 1990 to 4,722.6 (95% UI: 3,930.9-5,148.6) in 2021 (+7.4%). Scotland followed with an 8.1% rise, from 4,021.8 (95% UI: 3,465.6-4,636.8) to 4,348.8 (95% UI: 3,775.0-4,948.3). Wales showed a smaller change (+4.9%), from 3,950.1 (95% UI: 3,393.7-4,528.1) to 4,143.2 (95% UI: 3,614.4-4,741.1), while Northern Ireland had a 6.6% increase, from 3,993.2 (95% UI: 3,459.6-4,566.4) to 4,255.1 (95% UI: 3,702.2-4,880.1), as shown in Figure 2.

Sex-specific analyses revealed consistently higher prevalence among women. In England, female age-standardised prevalence rose from 4,804.7 per 100,000 (95% UI: 4,180.9-5,483.6) in 1990 to 5,226.8 (95% UI: 4,543.1-5,949.4) in 2021 (+8.8%). Scotland recorded an 8.9% increase, from 4,682.4 (95% UI: 4,044.1-5,411.6) to 5,100.2 (95% UI: 4,422.0-5,859.3). Wales rose by 6.9%, from 4,611.2 (95% UI: 3,996.7-5,265.7) to 4,931.1 (95% UI: 4,279.7-5,643.6), and Northern Ireland increased by 7.6%, from 4,645.7 (95% UI: 4,002.1-5,350.2) to 5,000.2 (95% UI: 4,344.8-5,704.6), as shown in Figure 2.

Among men, England maintained the highest rates, increasing from 3,465.8 (95% UI: 3,004.4-3,971.6) to 3,753.2 (95% UI: 3,252.0-4,283.3) (+8.3%). Scotland rose by 11.2%, from 3,154.7 (95% UI: 2,744.0-3,642.8) to 3,507.8 (95% UI: 3,023.1-4,043.9). Wales saw an 8.9% increase, from 3,124.9 (95% UI: 2,658.3-3,575.9) to 3,401.7 (95% UI: 2,926.9-3,912.9), and Northern Ireland had the smallest proportional rise (+9.3%), from 3,149.5 (95% UI: 2,687.6-3,628.6) to 3,442.3 (95% UI: 2,966.8-3,982.4), as shown in Figure 2.

Incidence

Between 1990 and 2021, the number of new knee OA cases increased markedly across all UK nations. In England, the incidence rose from 227,900 cases (95% UI: 199,000-262,000) to 334,600 (95% UI: 293,300-385,700), a 46.8% increase and the largest absolute growth among the nations. Scotland experienced a 43.0% rise, from 23,100 cases (95% UI: 19,900-26,600) to 33,000 (95% UI: 28,600-38,100). Wales recorded a 33.8% increase, from 13,600 cases (95% UI: 11,700-15,800) to 18,200 (95% UI: 15,900-21,000). Northern Ireland had the highest relative growth, rising 69.6% from 6,100 cases (95% UI: 5,400-7,100) to 10,400 (95% UI: 9,100-12,000).

**Figure 3:**
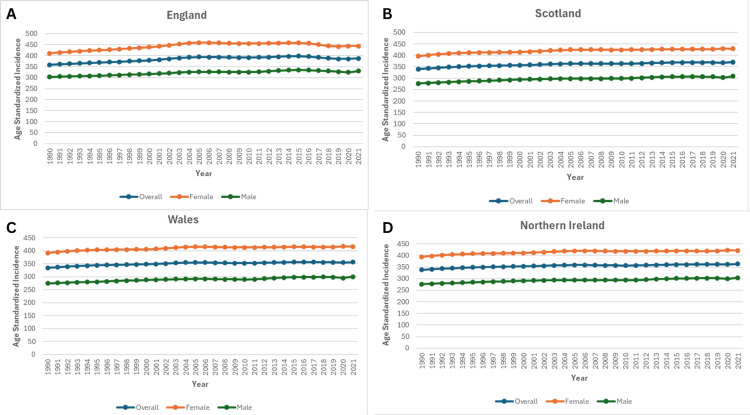
Trends in Age-Standardised Incidence of Knee Osteoarthritis in the United Kingdom, from 1990 to 2021 Panel A: Age-standardised incidence in England. Panel B: Age-standardised incidence in Scotland. Panel C: Age-standardised incidence in Wales. Panel D: Age-standardised incidence in Northern Ireland. Age-standardised incidence rates of knee OA in the United Kingdom from 1990 to 2021, stratified by country. The figure demonstrates temporal trends highlighting regional variations.

Age-standardised incidence rates also showed moderate increases across all four nations over the same period. In England, the rate rose from 358.5 per 100,000 (95% UI: 311.2-409.4) in 1990 to 387.5 (95% UI: 336.2-441.8) in 2021, an 8.1% increase. Scotland’s rate increased by 8.9%, from 339.6 (95% UI: 293.8-391.6) to 369.8 (95% UI: 322.2-422.8). Wales recorded a 6.6% rise, from 334.8 (95% UI: 290.3-385.7) to 356.8 (95% UI: 311.9-407.7), while Northern Ireland showed the smallest change (+7.6%), from 337.9 (95% UI: 293.6-392.3) to 363.7 (95% UI: 317.6-417.6), as shown in Figure 3.

The age-standardised incidence rates (ASIRs) of knee OA in the United Kingdom from 1990 to 2021 demonstrated distinct regional and sex-specific patterns. Among men, England consistently recorded the highest incidence, increasing from 303.34 in 1990 to 329.67 in 2021, representing an 8.68% rise. Scotland and Northern Ireland followed similar upward trajectories, with Scotland experiencing the largest proportional increase (11.45%, from 276.57 to 308.22). Wales exhibited a more moderate increase of 9.40% (from 274.61 to 300.35), as shown in Figure 3.

For females, ASIRs were substantially higher than those observed in males, highlighting a marked sex disparity. England again reported the highest rates, rising from 410.62 in 1990 to 443.10 in 2021 (7.91% increase), although the growth rate was surpassed by Scotland (8.05%, from 396.44 to 428.37) and Northern Ireland (7.10%, from 393.88 to 421.84). Wales showed a comparatively smaller increase of 6.36% (from 391.69 to 416.59), as shown in Figure 3.

Years lived with disability (YLDs)

**Figure 4:**
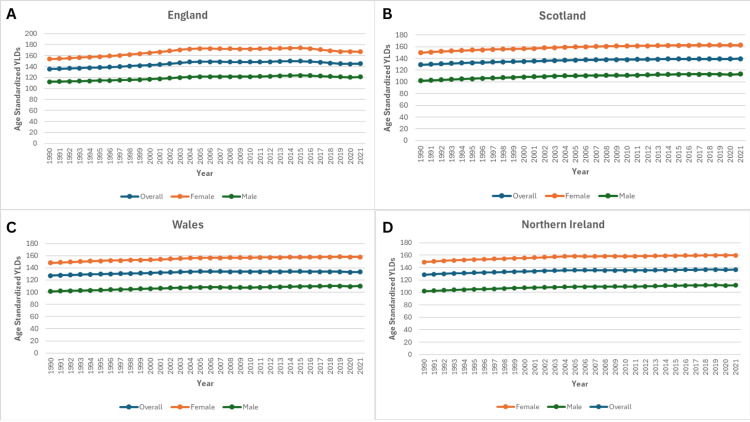
Trends in Age-Standardised YLDs in the United Kingdom, from 1990 to 2021 Panel A: Age-standardised YLDs in England. Panel B: Age-standardised YLDs in Scotland. Panel C: Age-standardised YLDs in Wales. Panel D: Age Standardised YLDs in Northern Ireland. Age-standardised YLDs rates of knee osteoarthritis in the United Kingdom from 1990 to 2021, stratified by country. The figure demonstrates temporal trends highlighting regional variations.

From 1990 to 2021, the age-standardised years lived with disability (YLDs) rates for knee OA increased across all four United Kingdom countries - England, Scotland, Wales, and Northern Ireland - though the magnitude of growth varied by region. In 1990, England reported the highest YLD rate (135.36), followed by Scotland (129.11), Northern Ireland (128.52), and Wales (127.33). By 2021, these values had risen to 145.05 (+7.16%), 139.36 (+7.94%), 136.81 (+6.45%), and 133.20 (+4.61%), respectively, with England maintaining the highest burden throughout the study period, as shown in Figure 4.

Sex-specific analysis revealed persistently higher YLD rates among women compared to men in all the regions. Among men, England again recorded the highest rates, increasing from 112.23 in 1990 to 121.22 in 2021 (+8.01%). Scotland exhibited the largest relative increase in male YLDs (+11.02%), rising from 102.08 to 113.33, while Wales and Northern Ireland experienced increases of 8.62% and 9.28%, respectively, as shown in Figure 4.

In women, baseline YLD rates in 1990 were substantially higher than in males, ranging from 148.21 in Wales to 153.93 in England. By 2021, England reached 166.92 (+8.44%), while Scotland and Northern Ireland demonstrated similar proportional growth. Wales showed the smallest increase (+6.39%) but still maintained markedly higher absolute rates compared to its male population, as shown in Figure 4.

Disability-adjusted life years (DALYs)

**Figure 5:**
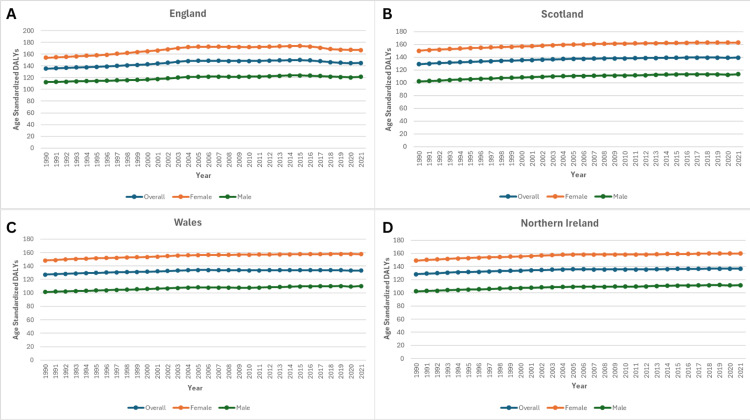
Trends in Age-Standardised DALYs in the United Kingdom, from 1990 to 2021. Panel A: Age-standardised DALYs in England. Panel B: Age-standardised DALYs in Scotland. Panel C: Age-standardised DALYs in Wales. Panel D: Age-standardised DALYs in Northern Ireland. Age-standardised DALYs rates of knee OA in the United Kingdom from 1990 to 2021, stratified by country. The figure demonstrates temporal trends highlighting regional variations.

From 1990 to 2021, age-standardised DALY rates for knee OA in the United Kingdom demonstrated consistent regional disparities and gradual overall increases. Among the total population, England persistently reported the highest rates, rising from 135.36 in 1990 to a peak of 149.94 in 2015 before declining slightly to 145.05 in 2021, representing a net increase of 7.16%. Scotland followed a similar upward trajectory, with rates increasing from 129.11 to 139.36 (7.94% increase), while Northern Ireland and Wales recorded more modest rises of 6.45% (128.52 to 136.81) and 4.61% (127.33 to 133.20), respectively, as shown in Figure 5.

When stratified by sex, women exhibited substantially higher DALY rates than men across all regions throughout the study period. In 1990, female DALY rates were highest in England (153.93), followed by Scotland (149.88), Northern Ireland (148.98), and Wales (148.21). By 2021, all regions recorded higher rates, with the largest relative increase observed in Scotland (8.6%, from 149.88 to 162.71) and the smallest in Wales (6.4%, from 148.21 to 157.68). England rose to 166.92 (8.4% increase), while Northern Ireland increased to 159.92 (7.3% increase), as shown in Figure 5.

The analysis of DALYs for knee OA in males across the United Kingdom from 1990 to 2021 demonstrates consistent increases in all regions, with notable variation in the magnitude of change. In 1990, England recorded the highest DALY rate at 112.23, followed by Northern Ireland (102.22), Scotland (102.08), and Wales (101.45). By 2021, DALY rates had risen in all regions, with Scotland showing the largest relative increase, from 102.08 to 113.33 (11.0%). Northern Ireland increased from 102.22 to 111.70 (9.3%), Wales from 101.45 to 110.19 (8.6%), and England from 112.23 to 121.22 (8.0%), the smallest proportional rise, as shown in Figure 5.

Trends in prevalence (Joinpoint analysis)

In Wales, from 1990 to 2005, the age-standardised prevalence showed a significant increase (APC=0.35%; 95% CI: 0.34 to 0.36; p<0.001). This was followed by a significant decline between 2005 and 2010 (APC=-0.09%; 95% CI: -0.16 to -0.02; p=0.019). From 2010 to 2015, the trend was non-significant (APC=0.05%; 95% CI: -0.02 to 0.12; p=0.170), before decreasing significantly again from 2015 to 2021 (APC=-0.08%; 95% CI: -0.12 to -0.04; p<0.001), as shown in Table 1.

**Table 1:** Joinpoint Regression Analysis for Trends in the Prevalence of Knee Osteoarthritis in the United Kingdom, from 1990 to 2021 *Asterisk indicates statistical significance at p<0.05. Annual percentage change (APC) values are based on Joinpoint regression analysis. p-values were calculated using the Monte Carlo permutation test. CI: Confidence interval.

Cohort	Segment	Lower Endpoint	Upper Endpoint	APC	Lower CI	Upper CI	Test Statistic (t)	Prob > |t|
Wales - 3 Joinpoints	1	1990	2005	0.35*	0.34	0.36	74.99	< 0.001
Wales - 3 Joinpoints	2	2005	2010	-0.09*	-0.16	-0.02	-2.55	0.019
Wales - 3 Joinpoints	3	2010	2015	0.05	-0.02	0.12	1.42	0.17
Wales - 3 Joinpoints	4	2015	2021	-0.08*	-0.12	-0.04	-4.12	< 0.001
Northern Ireland - 4 Joinpoints	1	1990	1994	0.50*	0.47	0.54	32.64	< 0.001
Northern Ireland - 4 Joinpoints	2	1994	2004	0.35*	0.34	0.36	78.95	< 0.001
Northern Ireland - 4 Joinpoints	3	2004	2011	-0.03*	-0.05	-0.01	-3.8	0.001
Northern Ireland - 4 Joinpoints	4	2011	2015	0.16*	0.11	0.21	6.59	< 0.001
Northern Ireland - 4 Joinpoints	5	2015	2021	0.06*	0.04	0.08	7.48	< 0.001
Scotland - 3 Joinpoints	1	1990	1994	0.59*	0.53	0.65	19.83	< 0.001
Scotland - 3 Joinpoints	2	1994	2004	0.36*	0.35	0.38	42.44	< 0.001
Scotland - 3 Joinpoints	3	2004	2015	0.13*	0.12	0.15	18.01	< 0.001
Scotland - 3 Joinpoints	4	2015	2021	0.04*	0	0.07	2.22	0.038
England - 6 Joinpoints	1	1990	1996	0.44*	0.43	0.45	69.14	< 0.001
England - 6 Joinpoints	2	1996	2001	0.73*	0.7	0.75	61.09	< 0.001
England - 6 Joinpoints	3	2001	2004	1.06*	0.97	1.14	27.98	< 0.001
England - 6 Joinpoints	4	2004	2011	-0.02*	-0.03	-0.01	-3.16	0.008
England - 6 Joinpoints	5	2011	2015	0.30*	0.26	0.34	16.11	< 0.001
England - 6 Joinpoints	6	2015	2019	-0.85*	-0.89	-0.81	-45.62	< 0.001
England - 6 Joinpoints	7	2019	2021	0.02	-0.06	0.1	0.56	0.585

In Northern Ireland, the prevalence of knee OA rose significantly between 1990 and 1994 (APC=0.50%; 95% CI: 0.47 to 0.54; p<0.001) and again from 1994 to 2004 (APC=0.35%; 95% CI: 0.34 to 0.36; p<0.001). A small but significant decline occurred between 2004 and 2011 (APC=-0.03%; 95% CI: -0.05 to -0.01; p=0.001), followed by significant increases from 2011 to 2015 (APC=0.16%; 95% CI: 0.11 to 0.21; p<0.001) and from 2015 to 2021 (APC=0.06%; 95% CI: 0.04 to 0.08; p<0.001), as shown in Table 1.

In Scotland, the prevalence increased significantly between 1990 and 1994 (APC=0.59%; 95% CI: 0.53 to 0.65; p<0.001) and from 1994 to 2004 (APC=0.36%; 95% CI: 0.35 to 0.38; p<0.001). The upward trend continued between 2004 and 2015 (APC=0.13%; 95% CI: 0.12 to 0.15; p<0.001) and persisted, albeit at a slower rate, from 2015 to 2021 (APC=0.04%; 95% CI: 0.00 to 0.07; p=0.038), as shown in Table 1.

In England, the prevalence rose significantly from 1990 to 1996 (APC=0.44%; 95% CI: 0.43 to 0.45; p<0.001) and from 1996 to 2001 (APC=0.73%; 95% CI: 0.70 to 0.75; p<0.001), followed by a sharp increase between 2001 and 2004 (APC=1.06%; 95% CI: 0.97 to 1.14; p<0.001). A slight but significant decline was observed from 2004 to 2011 (APC=-0.02%; 95% CI: -0.03 to -0.01; p=0.008), then a rise between 2011 and 2015 (APC=0.30%; 95% CI: 0.26 to 0.34; p<0.001). This was followed by a substantial decrease from 2015 to 2019 (APC=-0.85%; 95% CI: -0.89 to -0.81; p<0.001). The final segment, from 2019 to 2021, showed a non-significant change (APC=0.02%; 95% CI: -0.06 to 0.10; p=0.585), as shown in Table 1.

Trends in years lived with disability (YLDs): Joinpoint analysis

In Northern Ireland, the YLD rates increased significantly between 1990 and 1993 (APC=0.58%; 95% CI: 0.48 to 0.67; p<0.001) and continued to rise from 1993 to 2004 (APC=0.34%; 95% CI: 0.32 to 0.35; p<0.001). The period from 2004 to 2011 showed a non-significant change (APC=-0.02%; 95% CI: -0.06 to 0.01; p=0.126), followed by a significant increase between 2011 and 2018 (APC=0.13%; 95% CI: 0.10 to 0.17; p<0.001). From 2018 to 2021, the trend was again non-significant (APC=-0.01%; 95% CI: -0.11 to 0.08; p=0.780), as shown in Table 2.

**Table 2:** Joinpoint Regression Analysis for Trends in Knee Osteoarthritis Years Lived with Disability (YLDs) in the United Kingdom, from 1990 to 2021 *Asterisk indicates statistical significance at p<0.05. Annual percentage change (APC) values are based on Joinpoint regression analysis. p-values were calculated using the Monte Carlo permutation test. CI: Confidence interval.

Cohort	Segment	Lower Endpoint	Upper Endpoint	APC	Lower CI	Upper CI	Test Statistic (t)	Prob > |t|
Northern Ireland - 4 Joinpoints	1	1990	1993	0.58*	0.48	0.67	12.88	< 0.001
Northern Ireland - 4 Joinpoints	2	1993	2004	0.34*	0.32	0.35	48.5	< 0.001
Northern Ireland - 4 Joinpoints	3	2004	2011	-0.02	-0.06	0.01	-1.6	0.126
Northern Ireland - 4 Joinpoints	4	2011	2018	0.13*	0.1	0.17	8.85	< 0.001
Northern Ireland - 4 Joinpoints	5	2018	2021	-0.01	-0.11	0.08	-0.28	0.78
Wales - 4 Joinpoints	1	1990	1994	0.41*	0.33	0.5	10.04	< 0.001
Wales - 4 Joinpoints	2	1994	2000	0.27*	0.21	0.33	9.31	< 0.001
Wales - 4 Joinpoints	3	2000	2004	0.42*	0.28	0.56	6.47	< 0.001
Wales - 4 Joinpoints	4	2004	2018	0	-0.02	0.01	-0.62	0.544
Wales - 4 Joinpoints	5	2018	2021	-0.15*	-0.29	-0.02	-2.37	0.029
Scotland - 3 Joinpoints	1	1990	1994	0.59*	0.53	0.65	20.09	< 0.001
Scotland - 3 Joinpoints	2	1994	2004	0.36*	0.34	0.37	42.31	< 0.001
Scotland - 3 Joinpoints	3	2004	2015	0.14*	0.12	0.15	19.23	< 0.001
Scotland - 3 Joinpoints	4	2015	2021	0.01	-0.02	0.05	0.82	0.423
England - 6 Joinpoints	1	1990	1996	0.44*	0.43	0.46	61.98	< 0.001
England - 6 Joinpoints	2	1996	2001	0.68*	0.65	0.71	50.46	< 0.001
England - 6 Joinpoints	3	2001	2004	1.10*	1	1.19	25.79	< 0.001
England - 6 Joinpoints	4	2004	2011	-0.02*	-0.04	-0.01	-2.99	0.011
England - 6 Joinpoints	5	2011	2015	0.32*	0.28	0.37	15.29	< 0.001
England - 6 Joinpoints	6	2015	2019	-0.86*	-0.9	-0.81	-40.76	< 0.001
England - 6 Joinpoints	7	2019	2021	-0.09	-0.18	0.01	-2.05	0.063

In Wales, YLDs rose significantly from 1990 to 1994 (APC=0.41%; 95% CI: 0.33 to 0.50; p<0.001) and from 1994 to 2000 (APC=0.27%; 95% CI: 0.21 to 0.33; p<0.001), followed by another significant increase between 2000 and 2004 (APC=0.42%; 95% CI: 0.28 to 0.56; p<0.001). The trend plateaued from 2004 to 2018 (APC=0.00%; 95% CI: -0.02 to 0.01; p=0.544) and then declined significantly between 2018 and 2021 (APC=-0.15%; 95% CI: -0.29 to -0.02; p=0.029), as shown in Table 2.

In Scotland, YLDs increased significantly from 1990 to 1994 (APC=0.59%; 95% CI: 0.53 to 0.65; p<0.001) and from 1994 to 2004 (APC=0.36%; 95% CI: 0.34 to 0.37; p<0.001), with continued but slower growth between 2004 and 2015 (APC=0.14%; 95% CI: 0.12 to 0.15; p<0.001). From 2015 to 2021, the change was not statistically significant (APC = 0.01%; 95% CI: -0.02 to 0.05; p=0.423), as shown in Table 2.

In England, YLDs rose significantly between 1990 and 1996 (APC=0.44%; 95% CI: 0.43 to 0.46; p<0.001) and from 1996 to 2001 (APC=0.68%; 95% CI: 0.65 to 0.71; p<0.001). The most marked increase occurred from 2001 to 2004 (APC=1.10%; 95% CI: 1.00 to 1.19; p<0.001). A slight but significant decline followed between 2004 and 2011 (APC=-0.02%; 95% CI: -0.04 to -0.01; p=0.011) and then an increase from 2011 to 2015 (APC=0.32%; 95% CI: 0.28 to 0.37; p<0.001). This was followed by a substantial decline from 2015 to 2019 (APC=-0.86%; 95% CI: -0.90 to -0.81; p<0.001), and a non-significant decrease between 2019 and 2021 (APC=-0.09%; 95% CI: -0.18 to 0.01; p=0.063), as shown in Table 2.

Trends in age-adjusted disability-adjusted life years (DALYs): Joinpoint analysis

In England, DALY rates increased significantly from 1990 to 1996 (APC=0.44%; 95% CI: 0.43 to 0.46; p<0.001) and from 1996 to 2001 (APC=0.68%; 95% CI: 0.65 to 0.71; p<0.001). The steepest rise was observed between 2001 and 2004 (APC=1.10%; 95% CI: 1.00 to 1.19; p<0.001). This was followed by a slight but significant decline from 2004 to 2011 (APC=-0.02%; 95% CI: -0.04 to -0.01; p=0.011), an increase from 2011 to 2015 (APC=0.32%; 95% CI: 0.28 to 0.37; p<0.001), and a substantial decline from 2015 to 2019 (APC=-0.86%; 95% CI: -0.90 to -0.81; p<0.001). Between 2019 and 2021, the trend showed a non-significant decrease (APC=-0.09%; 95% CI: -0.18 to 0.01; p=0.063), as shown in Table 3.

**Table 3:** Joinpoint Regression Analysis for Trends in Knee OA in the United Kingdom, from 1990 to 2021 *Asterisk indicates statistical significance at p<0.05. Annual percentage change (APC) values are based on Joinpoint regression analysis. p-values were calculated using the Monte Carlo permutation test.

Cohort	Segment	Lower Endpoint	Upper Endpoint	APC	Lower CI	Upper CI	Test Statistic (t)	Prob > |t|
England - 6 Joinpoints	1	1990	1996	0.44*	0.43	0.46	61.98	< 0.001
England - 6 Joinpoints	2	1996	2001	0.68*	0.65	0.71	50.46	< 0.001
England - 6 Joinpoints	3	2001	2004	1.10*	1	1.19	25.79	< 0.001
England - 6 Joinpoints	4	2004	2011	-0.02*	-0.04	-0.01	-2.99	0.011
England - 6 Joinpoints	5	2011	2015	0.32*	0.28	0.37	15.29	< 0.001
England - 6 Joinpoints	6	2015	2019	-0.86*	-0.9	-0.81	-40.76	< 0.001
England - 6 Joinpoints	7	2019	2021	-0.09	-0.18	0.01	-2.05	0.063
Northern Ireland - 4 Joinpoints	1	1990	1993	0.58*	0.48	0.67	12.88	< 0.001
Northern Ireland - 4 Joinpoints	2	1993	2004	0.34*	0.32	0.35	48.49	< 0.001
Northern Ireland - 4 Joinpoints	3	2004	2011	-0.02	-0.06	0.01	-1.6	0.126
Northern Ireland - 4 Joinpoints	4	2011	2018	0.13*	0.1	0.17	8.85	< 0.001
Northern Ireland - 4 Joinpoints	5	2018	2021	-0.01	-0.11	0.08	-0.28	0.78
Scotland - 3 Joinpoints	1	1990	1994	0.59*	0.53	0.65	20.09	< 0.001
Scotland - 3 Joinpoints	2	1994	2004	0.36*	0.34	0.37	42.31	< 0.001
Scotland - 3 Joinpoints	3	2004	2015	0.14*	0.12	0.15	19.23	< 0.001
Scotland - 3 Joinpoints	4	2015	2021	0.01	-0.02	0.05	0.82	0.423
Wales - 4 Joinpoints	1	1990	1994	0.41*	0.33	0.5	10.04	< 0.001
Wales - 4 Joinpoints	2	1994	2000	0.27*	0.21	0.33	9.31	< 0.001
Wales - 4 Joinpoints	3	2000	2004	0.42*	0.28	0.56	6.47	< 0.001
Wales - 4 Joinpoints	4	2004	2018	0	-0.02	0.01	-0.62	0.544
Wales - 4 Joinpoints	5	2018	2021	-0.15*	-0.29	-0.02	-2.37	0.029

In Northern Ireland, DALYs rose significantly between 1990 and 1993 (APC=0.58%; 95% CI: 0.48 to 0.67; p<0.001) and from 1993 to 2004 (APC=0.34%; 95% CI: 0.32 to 0.35; p<0.001). From 2004 to 2011, the change was not statistically significant (APC=-0.02%; 95% CI: -0.06 to 0.01; p=0.126), followed by a significant increase from 2011 to 2018 (APC=0.13%; 95% CI: 0.10 to 0.17; p<0.001). The period from 2018 to 2021 showed a non-significant change (APC=-0.01%; 95% CI: -0.11 to 0.08; p=0.780), as shown in Table 3.

In Scotland, DALYs increased significantly between 1990 and 1994 (APC=0.59%; 95% CI: 0.53 to 0.65; p<0.001) and from 1994 to 2004 (APC=0.36%; 95% CI: 0.34 to 0.37; p<0.001). Growth continued, albeit at a slower pace, from 2004 to 2015 (APC=0.14%; 95% CI: 0.12 to 0.15; p<0.001). From 2015 to 2021, the change was not statistically significant (APC=0.01%; 95% CI: -0.02 to 0.05; p=0.423), as shown in Table 3.

In Wales, DALYs increased significantly between 1990 and 1994 (APC=0.41%; 95% CI: 0.33 to 0.50; p<0.001), from 1994 to 2000 (APC=0.27%; 95% CI: 0.21 to 0.33; p<0.001), and from 2000 to 2004 (APC=0.42%; 95% CI: 0.28 to 0.56; p<0.001). The trend remained stable between 2004 and 2018 (APC=0.00%; 95% CI: -0.02 to 0.01; p=0.544), followed by a significant decline between 2018 and 2021 (APC=-0.15%; 95% CI: -0.29 to -0.02; p=0.029), as shown in Table 3.

## Discussion

This study provides a comprehensive evaluation of temporal trends and regional disparities in the burden of knee OA across the United Kingdom from 1990 to 2021 using data from the GBD Study. Our findings show that over the past three decades, the prevalence, incidence, YLDs, and DALYs attributable to knee OA have consistently increased in all four UK countries. In terms of both absolute numbers and age-standardised rates, England carried the heaviest share of the national burden, with Wales, Scotland, and Northern Ireland showing different trends. The notable nonuniformity in the rate of increase raises the possibility of regional variations in reporting practices, healthcare access, lifestyle factors, and population demographics.

Our study's upward trend in knee OA burden is consistent with earlier national and international reports [1,6]. According to a 2017 GBD analysis, OA is one of the leading causes of disability globally, with knee OA accounting for the majority of OA-related disability [2]. Cross et al.'s UK-specific review and later NHS data analyses highlighted a consistent rise in the prevalence of OA, which was partly ascribed to an ageing population and rising obesity rates [2,18]. Similar increases have been reported abroad in high-income nations like Australia [19] and the United States [20], indicating a shared influence of risk factors related to lifestyle and demographics.

The observed regional disparities might be a reflection of variations in healthcare infrastructure, health behaviours, and socioeconomic status among the UK countries. For instance, compared to England, Wales and Scotland have historically had greater rates of obesity prevalence and physical inactivity, two major risk factors for knee OA [21,22]. On the other hand, England's greater absolute burden might just be a result of its bigger population and better ability to report diagnoses.

The increasing prevalence of knee OA in the UK is probably caused by a number of factors. First, population ageing plays a central role, as OA is strongly age-related, with prevalence increasing sharply after the fifth decade of life [8]. Therefore, the number of people at risk has increased due to the UK's ageing demographic profile. Second, because excess body weight raises the mechanical load on knee joints and triggers inflammatory pathways that hasten cartilage degradation, the obesity epidemic has had a major impact on OA trends [23]. Interestingly, since the early 1990s, the prevalence of obesity in the UK has more than doubled [24,25], which closely reflects the rising OA trends we observed. Third, lifestyle and work-related factors influence the course of the disease; sedentary habits reduce muscle strength and joint mobility, while some high-impact work activities encourage cartilage degradation [26]. The subnational variations observed in England, Scotland, Wales, and Northern Ireland may also be partially explained by differences in occupational exposure between rural and urban areas. Finally, over time, there may have been more cases detected and reported due to improved diagnostic capabilities and awareness, such as expanded use of imaging modalities, primary care screening programmes, and enhanced public knowledge of OA [27].

The growing burden of knee OA has profound implications for healthcare systems, workforce productivity, and overall quality of life. As one of the leading causes of disability and work absenteeism, OA results in substantial economic costs through increased healthcare utilisation, lost productivity, and disability benefits [28]. England's large absolute burden emphasises the urgent need for scalable, nationwide preventive efforts, whereas Wales and Scotland have considerably higher age-standardised rates, indicating the need for region-specific policies. Potentially beneficial approaches include population-level obesity prevention initiatives, promotion of physical activity regimens suited to joint health, and early detection of OA with conservative therapy to delay or prevent surgical intervention. Given that knee replacement surgery is the most popular elective procedure in the UK [29], attempts to reduce OA progression should result in significant healthcare cost reductions while improving patient outcomes.

This study has several limitations that should be considered when interpreting the findings. First, the research was based on secondary data from the GBD 2021 study, which combines information from many sources and employs statistical modelling to fill data gaps. While this strategy provides complete coverage, it increases possible ambiguity due to estimating methodologies and assumptions. Second, knee OA diagnoses in the GBD framework may rely on a combination of clinical assessments, self-reports, and administrative records rather than standardised radiological confirmation, increasing the risk of misclassification. Third, changes in case definitions, healthcare availability, and reporting processes between UK nations may have an impact on the observed regional variances. Fourth, the ecological design of this study makes causal inference impossible, and individual-level factors such as body mass index, physical activity, genetics, and comorbidities cannot be investigated.

## Conclusions

From 1990 to 2021, the burden of knee OA in the United Kingdom increased significantly, driven by population ageing, rising obesity incidence, lifestyle factors, and improved identification. While England has the most incidence of knee OA, greater age-standardised rates in Wales and Scotland reveal regional variations that require targeted interventions. Addressing modifiable risk factors, particularly obesity and physical inactivity, as well as early diagnosis and conservative management options, will be critical to slowing disease progression, lowering disability, and alleviating the mounting healthcare and economic burden. Sustained, coordinated public health strategies and resource allocation are essential for reversing present trends and promoting long-term musculoskeletal health in the UK.
